# The Regulatory Role of Lipid Metabolism in Endometrial Cancer

**DOI:** 10.1155/2022/6458877

**Published:** 2022-08-29

**Authors:** Shuyang Zhang, Xingyu Han

**Affiliations:** Medical College, China Three Gorges University, Yichang 443002, China

## Abstract

Endometrial cancer is the 6th most common carcinoma as well as the 2nd most common malignancy worldwide in women. It is closely related to fat content, and dyslipidemia is among the most significant metabolic changes in this cancer. Therefore, further understanding of the regulation mechanism in lipid metabolism of endometrial cancer is conducive to the development of better therapeutic strategies and methods. Here, we systematically review the signaling pathways that regulate lipid metabolism in endometrial cancer and the research progress of drugs and targeted therapies that act on lipid metabolism by retrieving relevant articles. The underlying mechanism of occurrence and development of endometrial cancer is relatively clear and comprehensively reviewed here. But following more research studies will help to illuminate more specific regulatory roles of lipid metabolism in endometrial cancer and explore new possible mechanisms, prognostic and therapeutic targets, and subsequent drugs. Our review will provide a full view for the following investigation of lipid metabolism in endometrial cancer.

## 1. Introduction

Endometrial cancer (EC) ranks 2nd among gynecological malignancies worldwide. In the United States, there were approximately 66,570 new cases in 2021, with 12,940 patient deaths [1]. In recent years, the incidence of endometrial cancer in our country has increased significantly. In 2020, about 81,964 new cases of endometrial carcinoma appeared in China, with 16,607 patient deaths [2, 3]. Associated risk factors include continued estrogen exposure (ovarian anovulatory dysfunction and estrogen-secreting ovarian tumors (estrogen replacement therapy without progesterone protection and selective estrogen receptor modulator therapy, such as tamoxifen)), metabolic abnormalities (such as obesity and diabetes), early menarche, infertility, delayed menopause, carrying genetic susceptibility genes for endometrial cancer, such as Lynch syndrome, Cowden syndrome, and polymerase proofreading-associated polyposis (PPAP), and advanced age [4, 5]. More than 90% of patients are in their 50s, with a median age of 63 at diagnosis, while 4% of patients were younger than 40 years old at diagnosis, and some patients have the will to preserve fertility [4, 6]. 80% of patients with endometrial cancer can be diagnosed at an early stage, with the tumor confined to the uterus, and the 5-year survival rate is greater than 95% [4]; if there is local spread or distant metastasis, the 5-year survival rate drops to 68% or 17%, respectively [7]. Factors associated with preventing endometrial cancer include parity and the use of oral contraceptives. Parity was inversely proportional to the risk of endometrial carcinoma [8]. The use of oral contraceptives can reduce the hazard of developing cancer in the womb by 30 to 40%, and the protective effect is longer lasting over time. Protection can even last decades after it is stopped [9].

The prognosis of endometrial cancer depends on the patient's age at diagnosis, the pathological stage and type, and the degree of tumor differentiation. The prognosis of patients with advanced age, advanced stage, and poor differentiation is worse [10, 11]. Clinically, endometrial cancer can be classified into type I/II (Bokhman classification) [12], and the former is hormone-dependent with mainly endometrioid carcinoma of the pathological type, accompanied with better prognosis, while type II is non-invasive and hormone-dependent type, mainly including serous carcinoma, clear cell carcinoma, carcinosarcoma, and so on, which have a poor prognosis. Currently, endometrial cancer is mainly treated by surgery, with radiotherapy and chemotherapy as the commonly used adjuvant therapy. The treatment plan should be made in consideration of the patient's age, pathological type and molecular type, clinical staging, and physical performance status. In recent years, with the development of clinical studies, targeted therapy and immunotherapy have also shown good curative effects on endometrial cancer. In addition, the widespread application of genetic testing not only provides a basis for the diagnosis of hereditary endometrial cancers such as Lynch syndrome but also guides molecular typing of endometrial cancer and the selection of targeted drugs.

Endometrial cancer has been on the rise in recent years, owing to the influence of a high-fat, high-calorie diet and a sedentary lifestyle. Compared with all other malignancies, the strongest association with obesity was endometrial cancer. According to the American Cancer Society's 2019 report, 57% of endometrial cancer patients are obese, and a significant correlation exists between BMI and endometage group. Obese postmenopausal women are 1.56 times more likely than non-obese women to suffer endometrial cancer. Perimenopausal obese women are twice as probably as norial cancer [13]. Women with a normal body mass index (BMI) have a 3% increased lifetime risk of endometrial cancer, and each 5-unit rise in BMI increases the risk by 50% [14, 15]. The association between obesity and the incidence of endometrial cancer varies by n-obese women to acquire endometrial cancer [16, 17]. According to research, deliberate weight loss or maintaining a healthy weight can considerably lower the incidence of endometrial cancer or improve the prognosis of endometrial cancer patients [18]. Endometrial cancer was 1.34 and 1.65 times more common in patients with raised total cholesterol and high-density lipoprotein, respectively, than in normolipidemic patients [19]. In conclusion, aberrant lipid metabolism is an important cause of endometrial cancer. Abnormal fat metabolism is one of the most significant metabolic changes in tumors. Through fat metabolism, cells obtain energy, signaling molecules and biofilm components to gain the microstructure for proliferation, survival, invasion, metastasis and influence of tumors and the treatment of cancer [20]. As one of the most common female reproductive system tumors, endometrial cancer deserves further in-depth understanding. Therefore, more in-depth and effective disease prevention measures and better therapeutic drugs need to be developed. Here, based on the new research progress on lipid metabolism-regulating endometrial cancer, we will comprehensively review the research progress on lipid metabolism and endometrial cancer signaling pathway, small molecule drugs, and targeted therapy, to provide information for subsequent research and provide a reference for clinical diagnosis and treatment.

## 2. Signaling Pathways in the Lipid Metabolism of Endometrial Cancer

### 2.1. MAPK Family Signaling Pathway

**Figure 1:**
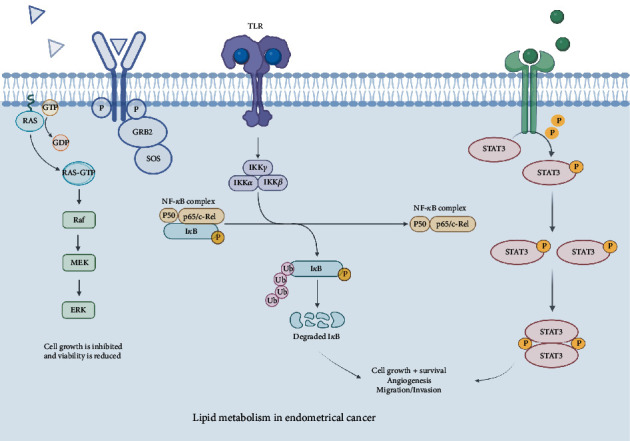
Signaling pathways and mechanisms in the lipid metabolism of endometrial cancer.

The signaling pathway of mitogen-activated protein kinase (MAPK) is diffusely expressed in many important cellular physiological and pathological processes (Figure 1). At the same time, it is one of the most frequently mutated oncogenic channels in malignant tumors. A large number of research studies have shown that the state of the MAPK pathway is frequently activated or altered in most cancers. MAPK signaling is triggered by the activation of a family consisting of small guanine triphosphatases (GTPases), which include RAS proteins (KRAS, NRAS, and HRAS) that integrate signaling from upstream to activate guanosine exchange factors (GEFs), causing downstream changes [21]. The MAPK which is mitogen-activated protein kinase pathways include MAPK, MAPK kinases (MKK and MEK) and MAPK kinase kinases (MKKK or MEKK). These three kinases can be successively activated and participate in the modulate of cell growth, differentiation, stress, inflammation and many other key physiological or pathological processes. The MAPK pathway has 4 major branching routes: ERK, JNK, p38/MAPK, and ERK5. In endometrial cancer-related studies, adiponectin treatment can downregulate ERK1/2 signaling, that is, through the ERK1/2-MAPK pathway, reducing cell viability and inhibiting cell proliferation in endometrial cancer cell lines [22]. In addition, adiponectin therapy inhibited the phosphorylation of AKT of KLE and HEC-1-A and inhibited the phosphorylation of ERK1/2 by expression and activation of PTEN gene [23]. Gal-3, Gal-3 in the galactocoagulin gene exists in a and concentration dependent form, and gal-3-induced ERK1/2 expression is also associated with calcium and protein kinase C-related activation [24, 25].

### 2.2. JAK-STAT Signaling Pathway

JAK kinases are a family of signaling molecules linked by intracellular structures of type I/II cytokine receptors and belong to the non-receptor class of tyrosine kinases (Figure 1). Constituents of the signal transducer and activators of transcription (STAT) protein family are pivotal proteins for cytokine signaling and interferon-related antiviral activity. These elements are able to transmit signals from the cell membrane to the nucleus, thus activating gene transcription. The JAK/STAT signaling pathway consists of ligand-receptor complexes, JAKs, and STATs. Through ligand-receptor binding, the receptor is dimerized, and the JAK coupled to the receptor is activated by autophosphorylation. The phosphorylated receptor recruits STAT, and then JAK phosphorylates STAT to form a dimer and enters the nucleus to regulate transcription and export signals. To date, we have identified four members of the JAK family: JAK1, JAK2, JAK3, and TYK2. The major STAT family members that have been discovered include STAT1, STAT2, STAT3, STAT4, STAT5a, STAT5b, and STAT6. Their signaling pathways are related to a variety of common cellular physiological processes, including proliferation, differentiation, apoptosis, angiogenesis, and immune system regulation [26].

In research studies related to lipid metabolism in endometrial cancer, omentin, an adipokine secreted from visceral fat, stimulates apoptosis by activating the JAK signaling pathway and p53 upregulation mechanism [25]. Leptin regulates PI3K/AKT3 and ERK1/2 signaling through the JAK/STAT pathway and increases the expression of anti-apoptotic proteins such as XIAAP and systemic inflammation factors (TNF-*α*, IL6). Meanwhile, increased expression of angiogenic factor (VEGF) and hypoxia-inducible factor-1a (HIF-1a) promotes endometrial cancer cell survival, proliferation, and migration [27].

### 2.3. NF-*κ*B/Notch1 Signaling Pathway

The NF-*κ*B family consists of five members, including p65 (RelA), RelB, c-Rel, p105/p50, and p100/p52 [28]. A number of illnesses and processes, including carcinoma, inflammatory and autoimmune diseases, septic shock, viral infection, and aberrant immunological development, have been linked to NF-B dysregulation. Ingredients of the I*κ*B kinase (IKK) complex, including the canonical heterotrimeric IKK*α*/IKK*β*/IKK*γ* and the alternative IKK*α*/IKK*α* homodimer, determine the activation pathway of NF-*κ*B (Figure 1). The Notch gene produces the Notch1 receptor, a single transmembrane protein. The Notch receptor, which controls cell differentiation, proliferation, and death, transmits signals between nearby cells. Through the NF-B/Notch1 signaling pathway, visfatin drives malignant behavior in the development of endometrial cancer and may stimulate cell proliferation, among other things. Another study found that vaspin reduced cancer cell motility and proliferation by blocking the NF-B/Notch1 signaling pathway [25, 29].

### 2.4. Reprogramming of Lipid Metabolism in Endometrial Cancer by ERR*α*

Under the condition of hypoxia and malnutrition, reprogramming of metabolism is the key to tumor survival. The stronger the metabolic plasticity of surviving tumor cells, the more malignant their biological behavior and the stronger the chemotherapy resistance [30]. HIF-1 regulates a wide variety of genes and proteins involved in cell metabolism, pH, and EMT, increasing the aggressiveness of tumor cells. ERR*α* expression is linked to both EC cell growth and death. HIF-1 transcriptional activity is increased in cancer cells, which promotes glucose and lipid metabolic remodeling. ERR promotes tumor cell adaptability to hypoxia by increasing glutamine metabolism and lipid de novo synthesis. In conclusion, HIF-1/ERR*α* interaction can promote tumor cell adaptability to hypoxia, increase cancer cell metabolism, and promote cancer cell resistance to pyrophosphorylation [31].

TFEB promotes EC migration via EMT signaling, regulates wound healing via TFEB-ERR in EK and ECC-1 cells, increases membrane fluidity, and promotes endometrial cancer cell invasion in TFEB-ERR axis-induced lipid reprogramming. Higher TFEB/ERR patients showed deeper myometrial infiltration and lower serum HDL values [32].

## 3. Other Regulators in the Lipid Metabolism of Endometrial Cancer

### 3.1. Research Progress of Other Molecules in Lipid Metabolism in Endometrial Cancer

Upregulation of ATP citrate lyase (ACLY), a sterol biosynthesis pathway enzyme, can encourage EC cell proliferation and colony formation while reducing apoptosis. Concurrent administration of drugs linked to obesity (estradiol, insulin, and leptin) promotes ACLY nuclear translocation through Akt-mediated phosphorylation of ACLY at Ser455. The nucleus-located ACLY promotes histone acetylation, which activates the DHODH and other pyrimidine metabolism genes. By focusing on the ACLY axis, new EC therapeutic strategies can be created and obesity risk factors can be linked to the control of histone acetylation [33].

The greatly elevated expression of monoacylglycerol lipase is closely connected with an increased risk of EC, and targeting MAGL could be a novel and effective treatment for EC. It is unclear how MAGL promotes tumorigenesis and growth in EC, and the possible mechanism and subsequent drug development need to be confirmed by further experiments in EC [34].

Altered choline phospholipid metabolism in endometrial cancer results from overexpression of choline kinase alpha and an overactivated deacylation pathway. Endometrial cancer has significantly dysregulated lipid metabolism, with increased phosphocholine levels being the most significant lipid-related change. A deacylation pathway that has been triggered, increased expression of the catabolic enzymes LYPLA1, LYPLA2, and GPCPD1, and increased expression of choline kinase alpha (CHKA) are thought to be the causes of the changes. After malignant transformation, CHKA is connected to endometrial hyperplasia, atypical hyperplasia, and adenocarcinoma tissue [35].

Cyclin-dependent kinase 8 (CDK8) is a member of the transcriptionally regulated CDK family, and in an *in vivo* mouse model, ectopic expression of CDK8 in KLE cells in endometrial cancer inhibits cell proliferation and effectively blocks tumor growth. In trans well, CDK8 overexpression in KLE cells inhibited cell migration and invasion. CDK8 deficiency increases lipid gene expression in endometrial cancer cells [36].

### 3.2. Small Molecule Drugs against Lipid Metabolism for Endometrial Cancer Therapy

Combined therapy with micronized estradiol and progesterone is a safe and effective conservative treatment for early-stage endometrial cancer (stage IA/G1) in patients with polycystic ovary syndrome who wish to preserve fertility. Simultaneous use of antidiabetic, antioxidant, antidopaminergic, and anti-serotonergic drugs helps restore female sex hormone concentrations and normal endometrium [37].

Silibinin reduces endometrial cancer cell growth, produces cell cycle arrest, and promotes cell death through reducing STAT3 phosphorylation expression. It may also control the expression of downstream genes engaged in cell cycle and death in EC cells, such as endogenous SREBP1, which reduces lipid buildup in EC cells [38].

Raloxifene, a selective estrogen receptor modulator, exerts estrogenic antagonism on bone and lipid metabolism, which helps to protect the endometrium and prevent endometrial cancer [39].

### 3.3. Other Targeted Therapies for Endometrial Cancer

Stearoyl-CoA desaturase 1 (SCD1) is a molecular target of many primary tumors. Studies have demonstrated that targeted knockdown of SCD1 can significantly inhibit endometrial cancer cell growth and induce cellular apoptosis *via* affecting the process of lipid metabolism. Targeted knockdown of short hairpin RNA and chemical inhibitors of SCD1 both inhibited foci formation in the metastatic endometrial cell line AN3CA. It can be seen that SCD1 is a potential therapeutic target for endometrial cancer and achieves the effect of anti-cancer therapy by inhibiting lipid metabolism in cancer cells [40].

## 4. Conclusion

The signaling pathways that regulate lipid metabolism in endometrial cancer include MAPK, JAK/STAT, NF-kB/Notch1, ERR*α* involved in lipid metabolism reprogramming, and other molecules including ATP citrate lyase (ACLY), monoacylglycerol lipase (MAGL), choline kinase alpha (CHKA), cyclin-dependent kinase 8 (CDK8), and so on. Specifically, adiponectin downregulated ERK1/2-MAPK signaling, decreased ERK1/2 phosphorylation in RL95-2 cells, and inhibited cell proliferation and migration. Leptin promotes cell proliferation and migration through the JAK/STAT pathway. Omentin activates the JAK signaling pathway to stimulate apoptosis. Vaspin inhibits cancer cell proliferation and chemotaxis by inhibiting NF-*κ*B/Notch1 signaling pathway. HIF-1/ERR connection can accelerate tumor cell tolerance to hypoxia and improve metabolism, whereas TFEB-ERR axis-induced lipid reprogramming improves membrane fluidity and promotes cell invasion. Upregulation of ATP citrate lyase promotes cancer cell proliferation and colony formation, and increased monoacylglycerol lipase could increase cancer risk. Moreover, enhanced expression of choline kinase alpha (CHKA) promotes endometrial hyperplasia and deterioration.

In the treatment of lipid metabolism in endometrial cancer, micronized estradiol and progesterone are combined to treat early endometrial cancer (IA/G1 stage), and silibinin can effectively inhibit the proliferation of cancer cells by inhibiting the expression of STAT3 phosphorylation. Raloxifene also exerts estrogenic antagonism on bone and lipid metabolism and protects the endometrium. Based on the above studies, therapeutic targets can focus on targeting the knockdown of SCD1, ACLY axis, and MAGL. The underlying mechanism of occurrence and development of endometrial cancer is relatively clear, but further research is needed to illuminate more lipid metabolism-associated targets for future precise therapy.

## Data Availability

The data used to support the findings of this study are included within the article.
